# Supplementary figures and images for: The large-scale blast score ratio (LS-BSR) pipeline: a method to rapidly compare genetic content between bacterial genomes
Source: PeerJ. 2014 Apr 1;2:e332. doi: 10.7717/peerj.332 (PMC3976120; doi:10.7717/peerj.332)

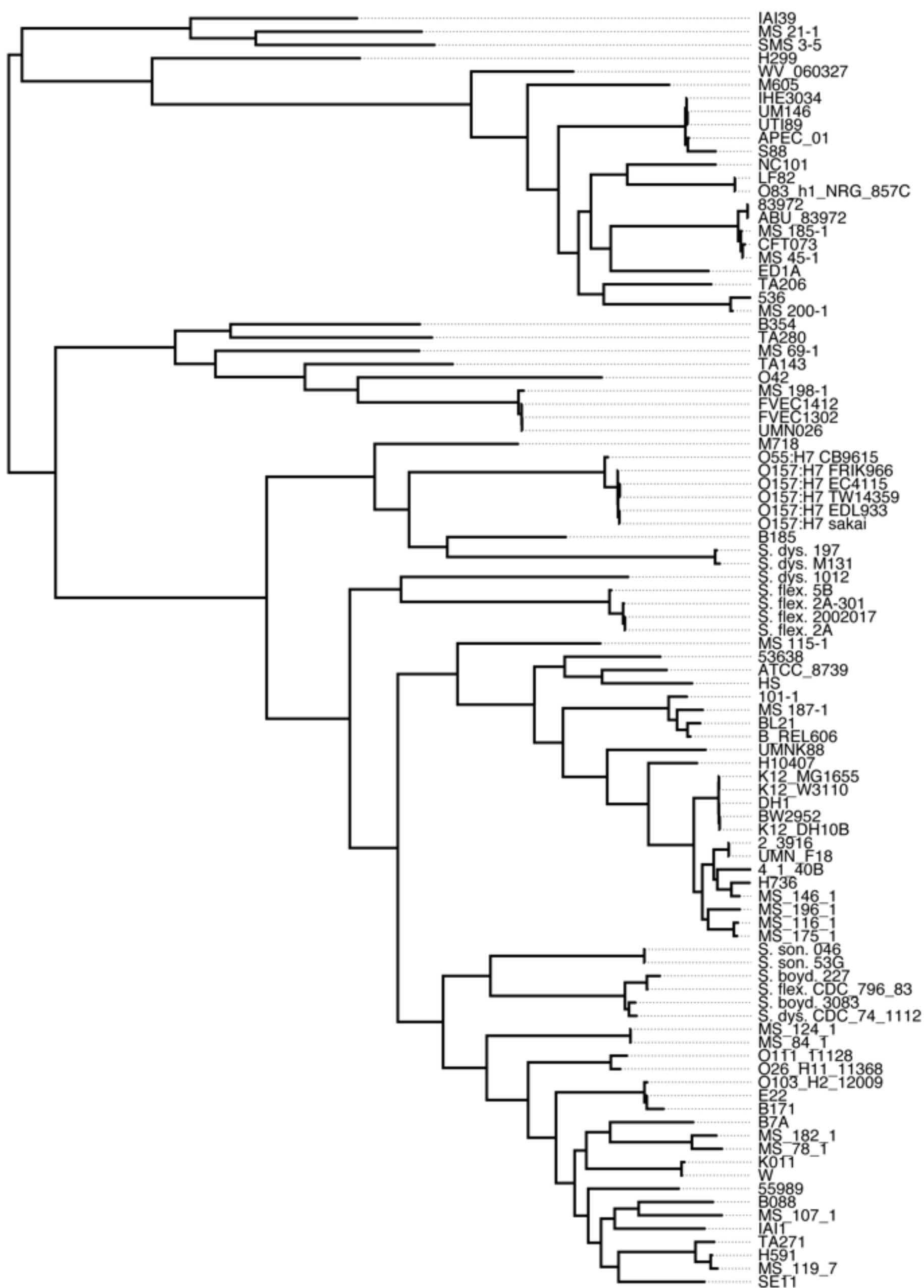

Supplement: Figure S1 — The core genome was extracted from the output of Mugsy (Angiuoli & Salzberg, 2011) and the phylogeny was inferred with FastTree2 (Price, Dehal & Arkin, 2010) . This phylogeny contains labels that can be used to identify specific genomes in Figs. 2 and 3. [file peerj-02-332-s001.pdf]
